# Nogo-A and Nogo-66 receptor in amyotrophic lateral sclerosis

**DOI:** 10.1111/j.1582-4934.2008.00351.x

**Published:** 2008-04-15

**Authors:** Felicia Yu Hsuan Teng, Bor Luen Tang

**Affiliations:** Department of Biochemistry, Yong Loo Lin School of Medicine, National University of SingaporeSingapore, Singapore

**Keywords:** amyotrophic lateral sclerosis, Nogo, Nogo-66 receptor, p75^NTR^

## Abstract

Nogo/reticulon (RTN)-4 has been strongly implicated as a disease marker for the motor neuron disease amyotrophic lateral sclerosis (ALS). Nogo isoforms, including Nogo-A, are ectopically expressed in the skeletal muscle of ALS mouse models and patients and their levels correlate with the disease severity. The notion of a direct involvement of Nogo-A in ALS aetiology is supported by the findings that Nogo-A deletion in mice reduces muscle denervation and prolongs survival, whereas overexpression of Nogo-A destabilizes motor nerve terminals and promotes denervation. Another intriguing, and somewhat paradoxical, recent finding revealed that binding of the Nogo-66 receptor (NgR) by either agonistic or antagonistic Nogo-66-derived peptides protects against p75 neurotrophin receptor (p75^NTR^)-dependent motor neuron death. Ligand binding by NgR could result in subsequent engagement of p75^NTR^, and this association could preclude pro-apoptotic signalling by the latter. Understanding the intricate interplay among Nogo isoforms, NgR and p75^NTR^ in ALS disease progression may provide important, therapeutically exploitable information.

## Introduction

Since its molecular identification, the adult central nervous system (CNS) myelin-associated Nogo [1–3] has been intensively investigated pertaining to its role in inhibiting CNS neuronal regeneration [4–6]. The *Nogo/reticulon (RTN)-4* gene has several splice isoforms, the major ones being Nogo-A, B and C. The longest of these, Nogo-A/RTN-4A, is enriched in the brain and spinal cord [7]. Nogo-B/RTN4B is found in the brain, but unlike Nogo-A, has a more ubiquitous expression pattern. Nogo-C/RTN-4C is particularly enriched in the muscle. The Nogo splice isoforms have N-terminal domains of varying length, but they share an identical C-terminal domain. This C-terminal domain, consisting of a 66-amino acid sequence (Nogo-66) flanked by two hydrophobic segments, is characteristic of the RTN family of proteins [8].

Molecular dissection of Nogo-A has revealed three separate regions that have neurite growth inhibitory activity [9, 10]. These include the N-terminal region encoded by exon 3, and the extracellular Nogo-66 segment. Nogo-66 binds to an axonal Nogo-66 receptor (NgR) [9]. The latter, a glycosylphosphatidylinositol (GPI)-linked molecule with leucine-rich repeats, also functions as a receptor for two other myelin-associated inhibitors, the myelin-associated glycoprotein (MAG) and the oligodendrocyte myelin glycoprotein (OMgp) [11–13]. A segment within the exon 3-encoded domain found at the N-terminus of Nogo-A also has a high affinity for NgR [14], and this could form a bipartite interaction with NgR, together with the Nogo-66 domain. Intriguingly, this segment could modulate the mode of binding of Nogo-66-derived peptides with NgR, effectively changing the downstream effect of the peptide ligand–receptor association. It is unclear if there exist other specific neuronal receptors for this exon 3-encoded domain of Nogo-A, but recent findings revealed that the N-terminal portion of Nogo-A acts through inhibition of the integrin signalling [15]. The very N-terminus of Nogo-A/B that is shared by both isoforms has also been associated with a vascular remodelling function upon injury [16], and a high-affinity receptor on the endothelial cells has been identified [17].

NgR requires membrane-spanning co-receptors to transduce growth inhibitory signals. The first NgR co-receptor identified is p75^NTR^[18, 19]. TAJ/TROY, an orphan TNF receptor family member broadly expressed during the development and in adult neurons, could serve as an alternative NgR co-receptor in place of p75^NTR^[20, 21]. Another membrane-spanning protein, LINGO-1 [22], may be necessary for the formation of a fully functional receptor complex that could transduce an inhibitory signal associated with Nogo-66-NgR binding. Even in nonneuronal cells, co-expression of LRR and Ig domain-containing, Nogo-Receptor-interacting protein (LINGO)-1 with NgR and p75^NTR^ is able to confer responsiveness to NgR ligand.

Other than being recognized as forming a major signalling axis inhibiting neuronal regeneration upon CNS injury, Nogo and NgR have been implicated in other neurological disorders [23]. Nogo-A is an important determinant of experimental autoimmune encephalomyelitis (EAE) development in experimental animals [24], and both Nogo and NgR are biomarkers for the corresponding human disease, multiple sclerosis [25–28]. Nogo-A levels have been shown to be markedly elevated in the hippocampal neurons of patients with temporal lobe epilepsy (TLE) [29]. Controversial evidence has also linked both Nogo-A and NgR to schizophrenia [30–34]. Nogo isoforms interact with the β-secretase beta-site APP-clearing enzyme (BACE)1 [35–37], while NgR was shown to bind the amyloid precursor protein [38]. Intriguingly, NgR antagonism reduces the brain amyloid load and improved memory in Alzheimer's disease (AD) transgenic models [39]. There is also an evidence for the elevation in Nogo-A [40] as well as NgR [41] levels in the hippocampal neurons of AD patients.

Another neurological disorder with a strong implication of Nogo involvement is amyotrophic lateral sclerosis (ALS), one of the most common forms of motor neuron degenerative diseases in adults [42]. Largely sporadic, ALS is characterized by a progressive atrophy of the skeletal muscle, paralysis due to degeneration of the brain and spinal cord neurons and subsequent death largely from neuromuscular respiratory failure. About 10% of ALS is hereditary in nature. Five genes inherited in a Mendelian manner are currently known to predispose an individual to typical ALS and ALS-like disorders [43], with a majority of familial ALS being traceable to missense mutations in the Cu/Zn-superoxide dismutase 1 (*SOD1*) gene [44, 45].

Recent findings have strongly implicated Nogo-A expression in the muscles as a potential biomarker for ALS [46–49], although other forms of myopathies may also have elevated muscle Nogo-A [50, 51]. The exact role of Nogo and its signalling in ALS patho-physiology is not yet clearly defined, but the muscle Nogo-A levels correlate with disease severity [52] and appear to be, at least, partly responsible for muscle denervation [53, 54]. Paradoxically, it was also found out very recently that NgR activation by Nogo-66-derived peptides appeared to confer a survival advantage to the motor neurons [55]. In the paragraphs below, we consider the neuropathological roles of Nogo and NgR in ALS in the light of these new findings.

## Alterations in Nogo isoform expressions in amyotrophic lateral sclerosis

The first association of Nogo with ALS was reported by Dupuis *et al.*[46]. The authors identified altered expression of Nogo iso-forms in the lumbar spinal cord of the *SOD1* (*G86R*) mutant transgenic mouse model of ALS using a subtractive hybridization approach. The examination of the gastrocnemius muscle of these mice revealed an anomalous, ectopically elevated expression of the transcripts of the adult brain-specific isoform Nogo-A. Nogo-B transcripts were also moderately elevated. On the other hand, the transcript levels of the adult muscle-enriched isoform Nogo-C were reduced. Experimentally induced denervation also reduced Nogo-C levels, but did not result in the ectopic elevation in Nogo-A. Elevated Nogo-A levels, to varying degrees, as well as depressed Nogo-C expressions, were also found in the postmortem and biopsy samples from diagnosed ALS patients, but not amongst control patients.

A further study by the authors on ALS patients indicated that Nogo-A and Nogo-B expression was significantly correlated with the disease severity measured by the ALS functional rating scale [52]. Nogo-A expression correlated, in particular, with the atrophy of the slow-twitch type 1 fibres, and almost all Nogo-A-positive fibres of this type appeared atrophic. These findings suggest that Nogo-A may be a useful marker to monitor ALS disease progression. A more recent study by the same authors appeared to have strengthened this notion. A proportion of individuals with lower motor neuron syndrome (LMNS), characterized by the presence of lower motor neuron signs (muscle weakness and atrophy with electromyographic abnormalities) but the absence of upper motor neuron signs, progressed to typical ALS. In a 1-year follow-up study, detection of Nogo-A in the muscle biopsy samples from LMNS patients identified the progression to ALS with a 91% accuracy, a 94% sensitivity and an 88% specificity [49]. Nogo-A may be detected as early as 3 months after the onset of LMNS symptoms for patients who eventually developed ALS. The muscle Nogo-A, therefore, has promising diagnostic and prognostic values.

The above view has recently been challenged by other workers. Wojcik and colleagues reported that the muscle biopsies from patients with other forms of myopathy and peripheral neuropathies also exhibited elevated Nogo-A immunoreactivity within the denervated muscle fibers [51]. Although the earlier study [46] showed that Nogo-A was not elevated in experimental muscle denervation, another report has detailed contradicting observations with the analysis of both Nogo-A transcripts and proteins [56]. These other reported observations may have weakened Nogo-A's potential as an ALS-specific marker [51, 57], and the merit of Nogo-A's diagnostic/prognostic value in ALS awaits further confirmation with larger clinical cohorts.

## Pathological roles of Nogo-A in ALS

Nogo may only be a bystander whose level changes in ALS, but does not influence the disease. The potentially more interesting possibility is that Nogo has a direct or indirect role in ALS progression, and that this could be exploited in a therapeutic sense. Nogo-C is enriched in the adult muscles, but its physiological role in the muscle function is unknown. As Nogo knockout mice depleted of all Nogo isoforms [58–60] have no apparent defects in the muscle function, it is more likely that the reduction in Nogo-C levels observed in ALS muscles is a consequence rather than a cause of the disease. Nogo-A, on the other hand, is well known as an inhibitor of neurite growth and axonal regeneration [4–6]. Its elevation in ALS muscles, therefore, makes the notion of its direct influence on the disease progression more intuitively likely.

Jokic *et al.* have recently provided evidence that the ectopic expression of Nogo-A in muscles contribute directly to the disease aetiology [53]. The authors crossed the ALS *SOD1* (*G86R*) mutant transgenic mice with a *Nogo-A*^−/−^ mutant mice [53] and showed that the resulting *G86R*/*Nogo-A*^−/−^ double-transgenic exhibited a moderate but significantly longer mean survival time compared to the *G86R* parent. These mice also have a higher number of motor neurons, reduced neuronal ubiquitin inclusions indicative of stress and a reduced expression of neuromuscular disease markers, acetyl-choline receptor and muscle-specific receptor of tyrosine kinase.

The authors have also asked if the ectopic overexpression of Nogo-A could induce a neuromuscular junction (NMJ) pathology. The muscle fibres transfected with a Nogo-A expression, constructed by electroporation, indeed exhibited aberrant synaptic structures and a significantly reduced postsynaptic structure size. Although it is unclear whether the levels of Nogo-A expression in these transient transfection experiments are comparable to those in ALS, and whether the overexpression of Nogo-B or Nogo-C has similar or different effects, these experiments suggest that the muscle fibre Nogo-A overexpression destabilizes NMJs. This destabilization may conceivably cause nerve terminal retraction and denervation, with consequential motor neuron death due to deprivation of target tissue neurotrophin, resulting in muscle atrophy. Synaptic destabilization by Nogo-A overexpression is, probably, not unique in NMJs and has also been observed in the cere-bellar Purkinje cells and their terminals [61].

## NgR- and p75^NTR^-mediated motor neuron death

Not all types of neurons are susceptible to growth inhibition by Nogo-A. The susceptibility would, necessarily, require the presence of the relevant receptors and signalling pathway components. It is also clear from recent findings that myelin-associated inhibitors signal growth inhibition through cell type-specific mechanisms [62, 63]. Another recent twist in the story of Nogo's involvement in ALS concerns its cognate receptor NgR and the co-receptor p75^NTR^. Dupuis and colleagues [55] showed that cultured embryonic motor neurons were susceptible to Nogo-66-induced growth cone collapse. The total neurite length was significantly reduced by Pep4, a peptide corresponding to the amino acids 31–55 of Nogo-66 and functioning as an NgR agonist [64], an effect that could be reversed by another peptide NEP 1–40 (amino acids 1–40 of Nogo-66), which functions as an NgR antagonist [64]. Both peptides, on their own, had no effect on the survival of the embryonic motor neurons under normal culture conditions and did not alter the course of neuronal death resulting from a growth factor (glial cell line-derived neurotrophic factor [GDNF]) deprivation. However, both peptides, surprisingly, rescued neuronal death induced by nerve growth factor (NGF) and nitric oxide (NO) as well as spinal cord extracts of *SOD1* (*G93A*) mice (another transgenic ALS model). This rescue was abolished by phosphatidylinositide-phospholipase C (PI-PLC) treatment (which cleaves GPI-linked proteins like NgR), a soluble dominant-negative NgR fragment, as well as antisense oligonucleotide-mediated silencing of NgR expression. Furthermore, the subcutaneous administration of these peptides could extend the survival of the spinal motor neurons *in vivo* after the sciatic nerve axotomy.

Although it is not yet shown if the administration of these peptides prolongs the motor neuron survival or general lifespan in ALS transgenic mice models, the findings outlined above are interesting and noteworthy in several aspects. First of all, NgR engagement by Nogo-66-derived peptides did not result in a general anti-apoptotic signalling, but rather in specifically countering p75^NTR^ mediated deaths. Apparently, it does not matter if the NgR is bound by an agonist or an antagonist. In other words, whether downstream signalling that leads to growth cone collapse as well as neurite growth inhibition was activated may be irrelevant in this case. What may be necessary is for the ligand-bound NgR to engage p75^NTR^ in a manner that precludes its apoptotic signalling. p75^NTR^ has multiple binding partners [65–67], and it is known that its formation of a receptor complex with sortilin mediates the apoptotic activity of the pro-forms of NGF and BDNF [68, 69]. It is possible that p75^NTR^'s interaction with the ligand-bound NgR binding could preclude or displace its interaction with sortilin. This notion is augmented by an interesting aspect of NgR signalling that has emerged recently – it is modulated by cell surface prote-olytic processing of itself and its co-receptor. The ligand binding to the cerebellar neurons induces α- and γ-secretase-mediated proteolytic cleavage of p75^NTR^ in a protein kinase C (PKC)-dependent manner [70]. The TNF-α converting enzyme (TACE)/a disintegrin and metalloprotease 17 (ADAM17) cleaves NgR and initiates intramembranous cleavage of p75^NTR^. In cultured dorsal root ganglion (DRG) neurons, this cleavage could underlie the attenuating effect of neurotrophic factors such as fibroblast growth factor 2 (FGF2) on the axonal growth inhibited by CNS myelin-associated inhibitors [71]. If the ligand-bound NgR does sequester a good fraction of p75^NTR^ away from the pro-apoptotic complexes, and its subsequent cleavage effectively precludes the pro-apoptotic signalling, this could be a part of the underlying mechanism of the motor neuron survival effects demonstrated by the Nogo-66-derived peptides. This speculation, however, needs to be confirmed experimentally.

That NgR ligand binding could enhance the survival of the motor neurons seems paradoxical in view of the earlier observations made by the same authors, which indicated that the absence of Nogo-A delays denervation, motor neuron death and disease survival [53]. An explanation offered by the authors is that known compensatory increase in Nogo-B expression in the knockout mice strain used [59] had provided the Nogo-66 required to elicit a pro-survival signalling against p75^NTR^-mediated motor neuron death. This explanation is speculative but entirely plausible as Nogo-A has an additional neurite growth inhibitory domain (encoded by the Nogo-A-specific exon 3) that is not present in Nogo-B [10]. A depletion in Nogo-A and an elevation in Nogo-B would, therefore, be beneficial for the motor neuron survival, particularly if the NMJ destabilization and nerve terminal retraction (and, therefore, neurotrophin deprivation) were mediated by the Nogo-A-specific inhibitory domain. This notion needs further experimental verification (in particular, whether Nogo-B overex-pression in the muscles has an effect that is opposite to Nogo-A).

It should be noted that the targeted disruption of NgR did not enhance the spinal cord neuron regeneration in some models [72], and recent studies suggest that it is required only for the acute growth cone collapsing of some neurons, but not for the chronic growth inhibitory actions of myelin inhibitors [73]. The Nogo-66–NgR axis may, therefore, not play a critical role in the destabilization of NMJ, and Nogo-66 may well function in a manner that is independent of NgR. The Nogo-A–NgR interaction on the motor neurons can also potentially be a complicated affair. The motor neurons themselves expressed significant amounts of Nogo-A [74], and these may engage the NgRs of the neighbouring neurons. As mentioned earlier, the Nogo-A-specific exon 3-encoded portion contains a domain that also binds NgR with nanomolar affinity and may interact with NgR in conjunction with Nogo-66 [14]. It is unclear at the moment if Nogo-A present in *cis* form with NgR on axons, or synapses, affects the latter's interaction in *trans* form with ligands from an opposing cell. It is also unclear as to how neuronal Nogo-A affects the interaction of NgR with its other ligands.

## Nogo and NgR as therapeutic targets for ALS?

Although Nogo-A's validity as a biomarker for ALS diagnosis/prognosis may need further confirmation, the above findings suggest Nogo and NgR could be potential targets for ALS adjunct therapy. This may be helped by the fact that the targeted deletion of both Nogo-A [58–60] and NgR [72, 75] in mice did not result in any clearly detectable defects. Therapeutic interventions resulting in their localized ‘inactivation’ would, therefore, be unlikely to incur severe side effects. A simple-minded postulation at this stage would suggest that Nogo-66-derived peptides (and other NgR-binding small molecules) could promote motor neuron survival, at least in the early stages of ALS. In this case, NgR antagonists may even have the dual effect of survival signalling as well as attenuation of Nogo-66-mediated denervation. On the other hand, targeting the Nogo-A-specific inhibitory domain, or localized Nogo-A silencing with siRNA, may help delay muscle denervation. However, much exploratory work lies ahead. Understanding the intricate interplay among Nogo, NgR and p75^NTR^ in normal and ALS disease settings would also be useful in other emerging non-CNS clinical conditions in which Nogo has been implicated, such as cardiomyopathy [76].
